# Microarray analysis identifies coding and non-coding RNA markers of liver injury in whole body irradiated mice

**DOI:** 10.1038/s41598-022-26784-w

**Published:** 2023-01-05

**Authors:** Molykutty J. Aryankalayil, Michelle A. Bylicky, Shannon Martello, Sunita Chopra, Mary Sproull, Jared M. May, Aman Shankardass, Laurel MacMillan, Claire Vanpouille-Box, Juan Dalo, Kevin M. K. Scott, C. Norman Coleman

**Affiliations:** 1 Radiation Oncology Branch, Center for Cancer Research, National Cancer Institute, National Institutes of Health, 10 Center Drive, Room B3B406, Bethesda, MD 20892 USA; 2 Gryphon Scientific, Takoma Park, MD 20912 USA; 3 Department of Radiation Oncology, Weill Cornell Medicine, New York, NY 10065 USA; 4 Radiation Research Program, National Cancer Institute, National Institutes of Health, Rockville, MD 20850 USA

**Keywords:** Non-coding RNAs, Biomarkers, Health care, Risk factors

## Abstract

Radiation injury from medical, accidental, or intentional sources can induce acute and long-term hepatic dysregulation, fibrosis, and cancer. This long-term hepatic dysregulation decreases quality of life and may lead to death. Our goal in this study is to determine acute changes in biological pathways and discover potential RNA biomarkers predictive of radiation injury. We performed whole transcriptome microarray analysis of mouse liver tissue (C57BL/6 J) 48 h after whole-body irradiation with 1, 2, 4, 8, and 12 Gray to identify significant expression changes in mRNAs, lncRNAs, and miRNAs, We also validated changes in specific RNAs through qRT-PCR. We used Ingenuity Pathway Analysis (IPA) to identify pathways associated with gene expression changes. We observed significant dysregulation of multiple mRNAs across all doses. In contrast, miRNA dysregulation was observed upwards of 2 Gray. The most significantly upregulated mRNAs function as tumor suppressors: *Cdkn1a*, *Phlda3*, and *Eda2r*. The most significantly downregulated mRNAs were involved in hemoglobin synthesis, inflammation, and mitochondrial function including multiple members of *Hbb* and *Hba*. The most significantly upregulated miRNA included: miR-34a-5p, miR-3102-5p, and miR-3960, while miR-342-3p, miR-142a-3p, and miR-223-3p were most significantly downregulated. IPA predicted activation of cell cycle checkpoint control pathways and inhibition of pathways relevant to inflammation and erythropoietin. Clarifying expression of mRNA, miRNA and lncRNA at a short time point (48 h) offers insight into potential biomarkers, including radiation markers shared across organs and animal models. This information, once validated in human models, can aid in development of bio-dosimetry biomarkers, and furthers our understanding of acute pathway dysregulation.

## Introduction

Radiation exposure from medical, accidental, or intentional events causes direct damage to DNA and production of reactive oxygen species (ROS), inducing further damage. Radiation injury to normal tissue may cause cell death and depletion of specific cell types, chronic redox stress leading to long-term dysfunction, and mutagenesis leading to oncogenesis^1,2^. Previous studies from atomic bomb survivors highlighted the impact of radiation on fatty liver development, long term liver dysfunction and cancer development^3,4^.

Toxicity and dysfunction in the liver have been reported extensively in the literature, with one study reporting that 70% (14/20) of patients displayed hepatic dysfunction as measured by dysregulation of liver transaminases and alkaline phosphatase levels after radiation therapy^5,6^. Development of fatty liver disease and insulin resistance has been observed at an increased rate in patients who received radiation therapy and in atomic bomb survivors^4,7^. Hepatic steatosis, the accumulation of fat in the liver, has also been reported in both rat and rabbit livers between two and six weeks after radiation injury, with greater severity of steatosis being associated with animal death^8–10^. Data from mini pigs indicated that 14 Gy localized radiation to the liver caused alterations in hepatocystolic function^11^. One study showed serum triglyceride and cholesterol levels increased in the livers of rats who received 6 Gy of radiation^12^.

Radiation-induced liver disease (RILD) is a dose limiting factor in radiation therapy to the abdomen that can develop in patients weeks to months after cessation of treatment^13^. The progression to RILD begins with tissue damage and endothelial cell death, which leads to inflammation and increased expression of cytokines as the liver attempts to repair itself^14,15^. The repair process includes proliferation of hepatocellular progenitors and myofibroblastic hepatic stellate cell transformation which can lead to fibrosis of the liver and RILD^16^. RILD symptoms include increased levels of liver enzymes including alkaline phosphatase, ascites, abdominal pain, and destruction of central veins. This destruction decreases oxygen delivery and causes tissue dysfunction, which can lead to death.^17,18^.

Understanding early alterations in transcription after radiation injury will provide new insights into strategies to prevent and mitigate normal liver damage. Further, while tools for radiation biodosimetry exist such as analysis of chromosomal damage, these assays to determine radiation dose may require a high level of technical experience, have low throughput, and can display low intercomparison accuracy^19^. Further research to develop a high throughput, accurate, and non-invasive method for radiation biodosimetry is ongoing. Prior research by others and from our own laboratory have highlighted the potential utility of messenger RNA (mRNA), microRNA (miRNA), and long non-coding RNA (lncRNA) as biomarkers for radiation injury and radiation sensitivity^20–22^.

Long non-coding RNA (lncRNA), RNA strands over 200 nucleotides that do not code for a protein, have previously been proposed as biomarkers for cancer, cardiovascular disorders, and other diseases^23,24^. Research from our lab and others have highlighted the radiation response of multiple lncRNA. Some of these lncRNA are otherwise uncharacterized, while others, such as *Trp53cor1* and *Dino*, are relatively well known^25–28^. MicroRNA (miRNA), non-coding RNA of roughly 22 nucleotides, exert their functions by hybridizing to complementary sequences in the 3’ UTRs of mRNAs, which lead to either RNA degradation or translation inhibition^29^. In addition, miRNA are stable in biofluids, making them a potentially useful prognostic and diagnostic indicator of radiation damage through minimally invasive blood draws^30,31^. Research to discover lncRNA-miRNA-mRNA networks and altered pathways after radiation in the lung has provided insights into early signs of dysfunction and potential biomarkers^32^.

We observed alterations in lncRNA and miRNA after radiation injury, which demonstrate their potential utility as part of a panel of RNA biomarkers to help determine radiation dose and potential pathogenesis^28,33^. This study will outline radiation induced dose dependent, liver specific, gene expression level changes after whole body irradiation (WBI) of mice. Understanding how radiation alters mRNA, lncRNA, and miRNA expression at 48 h will aid in identifying and predicting organ damage at both short- and long-term time points. It will also aid in developing strategies for mitigation of liver injury.

## Results

### Microarray analysis indicates an overall decrease in mRNA expression in liver after irradiation

**Figure 1:**
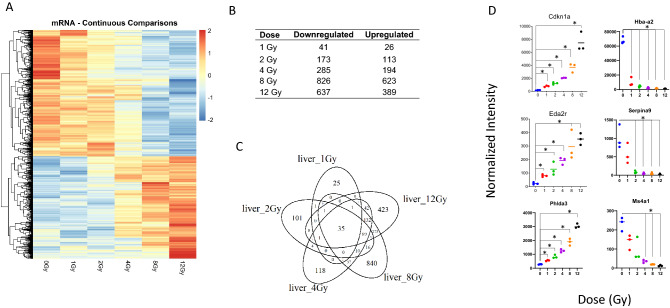
Radiation-induced gene expression profiles in mouse liver tissue. Whole genome microarray analysis was performed on all samples. A linear model was fit to each probe to evaluate differential expression of irradiated samples compared to controls. Criteria of (log_2_ fold change ((log2FC) > ^1^ and Benajmini-Hochberg adjusted (B-H) *p* value < 0.05) relative to controls were used to determine significance and differential expression. (**A**) Heatmap displays expression patterns, represented by z-score, of all differentially expressed mRNAs across all doses and controls. (**B**) Venn diagram shows dose distribution and overlap of differentially expressed mRNAs across all doses. (**C**) The number of down-regulated versus up-regulated mRNAs at each dose are shown in the table. (**D**) Examples of significant linearly up- and down-regulated mRNAs are shown to display the dose response to radiation in liver tissue samples. Asterisk (*) indicates statistical significance.

In total, analysis of microarray data (|log_2_FC|> 1; *p* value < 0.05) indicated that 2483 genes were shown to be differentially expressed between unirradiated control samples and at least one dose for WBI mice (Fig. 1A). Furthermore, microarray analysis from mouse liver tissue indicated that, at each dose of radiation, more mRNAs were downregulated than upregulated (Fig. 1B). Across all doses, 35 genes were dysregulated compared to control samples. In contrast, 25, 101, 118, 840, and 423 were exclusively expressed at 1, 2, 4, 8, and 12 Gy, respectively (Fig. 1C). Among all doses, the most significantly upregulated genes included *Cdkn1a, Eda2r*, and *Phlda3*, and the most significantly downregulated genes included *Hba-a2, Serpina9*, and *Ms4a1* (Fig. 1D). In addition to *Hba-a2*, we also observed significant downregulation of *Hba-a1, Hbb-b1, Hbb-b2, Hbb-bt* at all doses of radiation (Supplemental Fig. 1). These upregulated genes (*Cdkn1a, Eda2r* and *Phlda3*), are known for their role in cell cycle arrest, apoptosis and tumor suppression, while the most downregulated genes (*Hba-a2, Serpina9, and Ms4a1*) are associated with hemoglobin synthesis, mitochondrial function and B cell differentiation (Table 1)^34–43^. Supplemental Table S1 lists all genes dysregulated by radiation dose focusing on *p* value and log change. Further analysis of this data indicated that 1023 probes displayed a significant up or downregulation across all doses (Supplemental Table S1), with the top 20 and bottom 20 presented (Supplemental Table S2).

**Table 1:** Biological roles of most significantly dose-responsive mRNAs.

Gene Symbol	Gene Name	Biological Process Involvement	Previous report related to radiation
Cdkn1a	Cyclin Dependent Kinase Inhibitor 1A	Induces cell cycle arrest, apoptosis or senescence	PMID: 31857640, PMID: 26343536
Eda2r	Ectodysplasin A2 Receptor	Induces apoptosis	PMID: 27387861
Phlda3	Pleckstrin Homology Like Domain Family A Member 3	Tumor suppresser, AKT activity inhibitor	PMID: 19203586, PMID: 31046668
Serpina9	Serpin peptidase inhibitor, clade A, member 9	B cell development	PMID: 31105479
Ms4a1	Membrane spanning four domains subfamily a member 1	Encodes B lymphocyte antigen, aids in B cell differentiation, regulates calcium influx	PMID: 26620220
Hba-a2	Hemoglobin alpha, adult chain 2	Hemoglobin production, mitochondrial function	PMID: 18049034, PMID: 32957660

Genes displayed correspond to the top three most significantly dose-responsive up- and down-regulated mRNAs shown in Fig. 1D. A short-list of biological process involvement and previous reports of involvement in the molecular response to radiation are shown.

We sought to divide gene dysregulation into low (1 and 2 Gy), middle (4 Gy) and high (8 and 12 Gy) dose clusters (Fig. 1C). The low dose cluster had only one gene dysregulated in both 1 Gy and 2 Gy, Set, a nuclear proto-oncogene associated with DNA repair^44^ (Fig. 1C, Supplemental Table S1). In contrast, 8 Gy and 12 Gy share 273 genes which are not shared with the lower doses.

### Ingenuity pathway analysis of dysregulated genes highlights downregulation of immune response and increased cell cycle arrest

**Figure 2:**
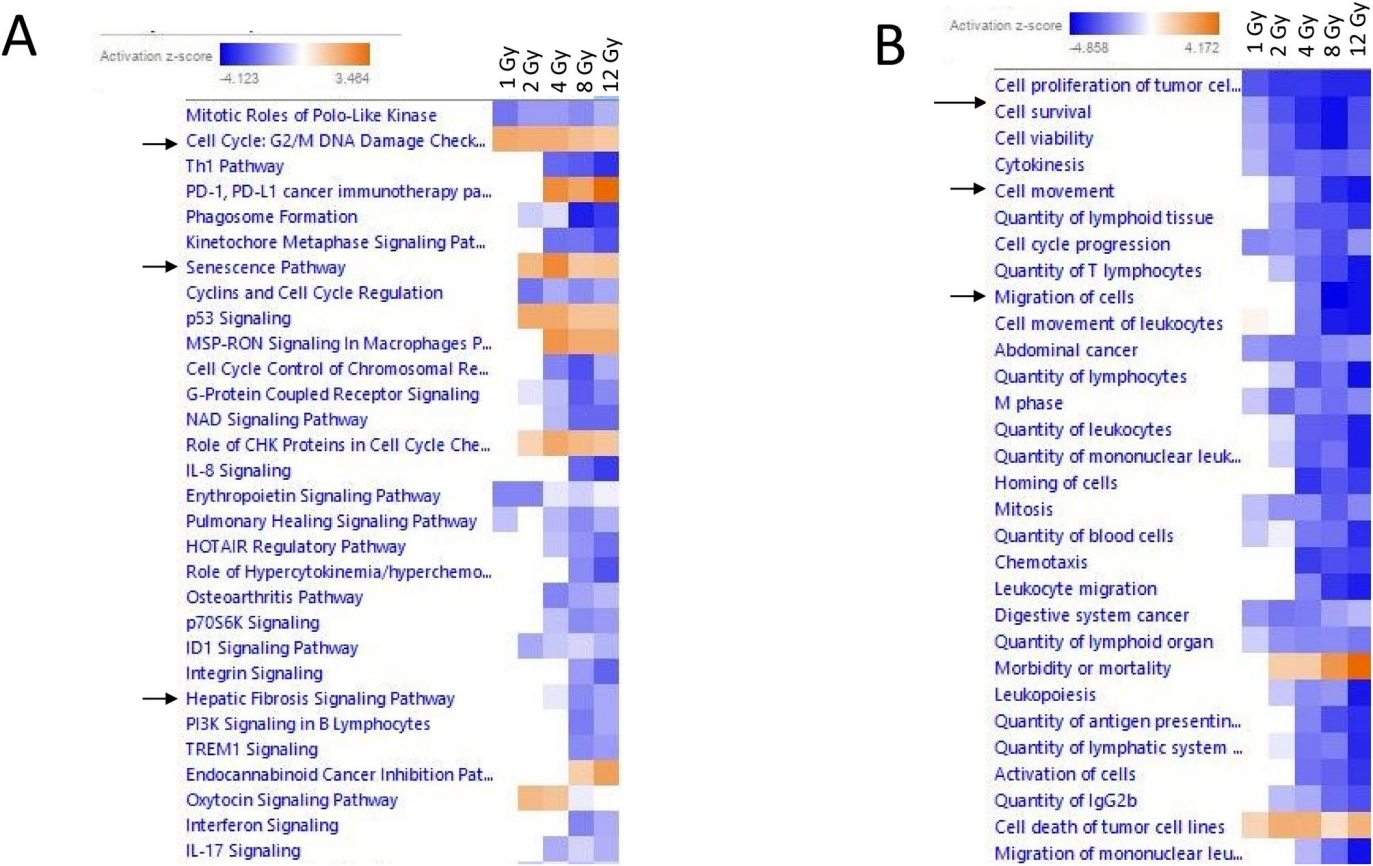
Predicted canonical pathway dysregulation in mouse liver samples based on all differentially expressed mRNAs. IPA was used to perform pathway analysis on all differentially expressed mRNAs to predict pathway involvement, independent of the target relationship with differentially expressed miRNAs. (**A**) Displays the top 30 most significantly dysregulated pathways (B-H *p* value < 0.05). A positive z-score indicates predicted activation of the pathway based on gene expression and a negative z-score indicates predicted deactivation of the pathway based on gene expression. Pathways are hierarchically clustered by z-score. (**B**) Displays top 30 most dysregulated functions (B-H *p* value < 0.05).

A canonical pathway analysis was performed with differentially expressed mRNA using IPA. The top 30 canonical pathways dysregulated by radiation and relevant to normal liver are presented in Fig. 2A (log_2_FC > 1, B-H *p* < 0.05) and the top 30 most dysregulated functions are in Fig. 2B. The only pathways altered at 1 Gy radiation were relevant to mitosis and erythropoietin signaling. Multiple pathways relevant to immune response were downregulated starting at 2 Gy radiation (Fig. 2A). Similarly, pathways relevant to senescence and cell cycle arrest were recruited starting at 2 Gy. Surprisingly, the hepatic fibrosis signaling pathway was downregulated at 4 Gy and continued to show downregulation at 8 and 12 Gy (Fig. 2A). Cell survival and cell migration pathways were downregulated as radiation dose increased (Fig. 2B).

We then divided these doses into low (1 and 2 Gy), middle (4 Gy), and high (8 and 12 Gy). We focused on the functions that were most severely activated based on Z-score from Fig. 2 to examine specific genes in the pathway or function. Our goal was to locate potential biomarkers which may be used to predict subsequent pathway dysfunction to aid in medical decision making. The observation of genes going from the high (Supplemental Fig. S2C) to middle (Supplemental Fig. S2B) to low (Supplemental Fig. S2A) are as follows. In 8 and 12 Gy samples, several downstream pathways relevant to inflammation were strongly upregulated. We present genes that IPA has associated with Inflammation of Body Cavity pathway based on 8 Gy (Supplemental Fig. S2C). Genes only dysregulated at 8 and 12 Gy include *Epha2, Il1r1, Lipin1, Cd40, Irf5, Gatm, Soat1, and Zbp1*. In 4 Gy samples, Apoptosis pathways were most strongly activated (Supplemental Fig. S2B). Genes only dysregulated at 4 Gy include *Ncam1, Brca2, Grb10, Ins1, Rcan2, Six4, Psme4 and Wsb1*. Oddly, while senescence pathways did not appear significant at 1 Gy within the comparison analysis, they were some of the most strongly activated pathways for 1 Gy when observed individually, with a Z-score greater than 2 for Senescence of Cells (Supplemental Fig. S2A). Within this senescence pathway most genes were also upregulated by all higher doses. Two genes were specific to a single dose. *Rad9b*, a checkpoint control protein, was only significant at 2 Gy^45^. *Wnt16*, which was previously shown to protect cartilage by inhibiting excessive WNT signaling in a mouse model, was only significant at 1 Gy^46^.

### Significant dysregulation is observed across solute carrier families as well as phase I and phase II metabolism genes in the liver

**Figure 3:**
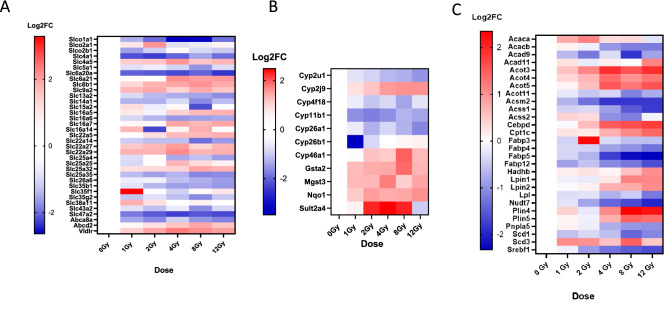
Drug and fat metabolism are dysregulated by radiation injury. Heatmaps of solute carriers (SLC) (A), phase I and phase II metabolism genes (B), and fat metabolism (C) are displayed. Only genes which were shown to be significantly altered ((log2FC > 1), *p* < 0.01) in at least one dose are displayed. White indicates control samples. Red indicates upregulation compared to control while blue indicates downregulation compared to control.

The liver’s role in detoxification and drug metabolism are well studied. We wanted to clarify how radiation impacts influx, phase I and phase II metabolism, and efflux of metabolites in the liver. We observed a decrease in solute carrier organic anion family (SLCO) genes, *Slco1a1, Slco2a1, Slco2b1* (Fig. 3A). We also observed decreased expression of solute carrier family (Slc) genes *Slc4a1, Slc5a1, Slc6a20a, Slc13a2*, and *Slc14a1*. Interestingly, families of solute carriers did not show consistent up or downregulation. While *Slc16a6* decreased in expression after radiation, *Slc16a5, Slc6a21,* and *Slc16a7* showed significant increases in expression by 8 Gy. Similarly, Slc22 family members *Slc22a5, Slc22a27, Slc22a29* also showed increased expression, with *Slc22a27* and *Slc22a29* showing statistical significance at 4 Gy while *Slc22a5* showed significance only at 12 Gy. In contrast *Slc22a14* showed significantly decreased expression at 8 Gy. *Slc25a25* showed significantly increased expression only at 4 Gy while *Slc25a35* was significantly decreased at 8 and 12 Gy. *Abca8a* was significantly decreased at 2, 8 and 12 Gy but not 4 Gy. While *Abcd2* was only upregulated significantly at 8 Gy. *Slc35b1* was decreased at 4 and 12 Gy while *Slc35g2* was only decreased at 8 Gy.

Due to the role of cyp450 genes in phase I metabolism we chose to study their expression across radiation doses (Fig. 3B). We separated genes in the heatmaps between genes relevant to transport (Fig. 3A), from genes relevant to metabolism of xenobiotics and biosynthesis (Fig. 3B) from genes relevant to fat metabolism (Fig. 3C). We observed dysregulation of several phase II metabolism genes including *Sult2a4, Gsta2*, and *Mgst3*. *Cyp26a1* showed a statistically significant decrease in expression at 12 Gy. *Cyp11b1* was significantly decreased at 2 and 4 Gy. *Cyp26b1* is significantly decreased only at 1 Gy. Interestingly, *Cyp2u1* was significantly decreased at 8 and 12 Gy while *Cyp2j9* was significantly increased at 8 and 12 Gy. *Cyp46a1* and the phase II metabolism gene *Gsta2* were only significant at 8 Gy. The phase II gene *Sult2a4* showed significantly increased expression at 4 and 8 Gy. *Mgst3* was significantly increased at 4 Gy. Schematic of radiation induced changes to transporters, Phase I and Phase II genes in liver are presented and further explained in the discussion (Supplemental Fig. S3A).

Since we observed changes to both active and passive transporters relevant to lipid metabolism and because there is a known link between fatty liver development and radiation, we chose to further study genes relevant to these pathways. *Acaca* shows a significant increase in expression at 2 Gy before decreasing to normal expression (Fig. 3C). *Acacb* showed decreased expression at 8 and 12 Gy. *Acad* members 9 and 11, *Cpt1c*, and *Hadhb* showed significant increases at 12 Gy. *Acot* family members 3, 4, and 5 showed significantly increased expression from 4 to 12 Gy. *Acsm2*, *Acss1*, and *Acss2* were all downregulated as dose increased. The genes *Cebpd*, *Srebf1*, *Lpin1*, *Lpin*2, *Plin4*, and *Plin5* showed increased expression with increasing dose. The *Fabp* family members 3, 4, 5, and 12 decreased with increasing radiation doses. *Scd1* decreased with increased radiation dose, while *Scd3* increased in expression with radiation dose.

In sum, we see a decrease in fatty acid uptake into the liver and a decrease in peroxisomal fatty acid oxidation as radiation doses increase. We also observe increased triacylglycerol synthesis and maintenance after 8 and 12 Gy doses of radiation as depicted in Supplemental Fig. S3B. Hepatic steatosis has previously been associated with RILD and death in animal models. Understanding the genes within this pathway are useful in developing biomarkers to predict liver metabolism dysfunction and greater liver damage.

### Increased dose of radiation caused increases in miRNA dysregulation

**Figure 4:**
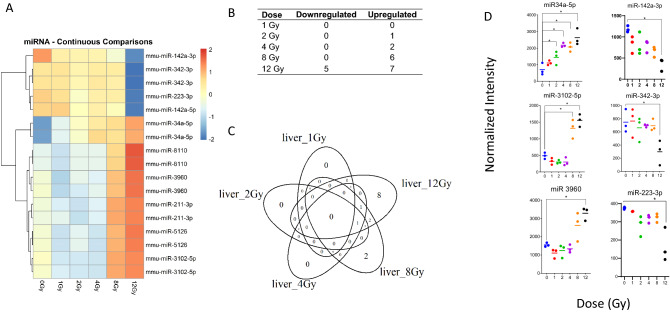
Radiation-induced microRNA expression profiles in mouse liver tissue. Microarray analysis was performed for all samples, and a linear model was fit to each miRNA probe to assess differential expression of irradiated samples compared to controls. Criteria of |log_2_FC|> 1 and B-H *p* value < 0.05 relative to controls were used to determine significance and differential expression. (**A**) Heatmap displays expression patterns, represented by z-score, of all differentially expressed miRNAs across all doses and controls. (**B**) The number of down-regulated versus up-regulated miRNAs at each dose are shown in the table. (**C**) Venn diagram shows dose distribution and overlap of differentially expressed miRNAs across all doses. (**D**) Examples of significant linearly up- and down-regulated miRNAs are shown to display the dose response to radiation in liver tissue samples. Asterisk (*) indicates statistical significance.

A separate whole genome analysis was performed on miRNA, (Fig. 4A). No miRNAs were significantly dysregulated at 1 Gy, while 12 miRNAs were dysregulated at 12 Gy (Fig. 4B). From 2 to 12 Gy, miRNA-34a-5p was significantly upregulated. In both 2 Gy and 4 Gy animals, only miR-34a-5p was upregulated. At 8 Gy miR-34a-5p, miR-3102-5p, 466n-3p and miR-302a5p were dysregulated. At 12 Gy miR-34a-5p, miR-3102-5p, miR-142a-5p, miR-142a-3p, miR-342-3p and miR-3960 were dysregulated (Fig. 4C). Notably, miR-466n-3p and miR-302a-5p were only dysregulated at 8 Gy. The most upregulated miRNA included miR-34a-5p, miR-3102-5p, and miR-3960, and the most downregulated included miR-142a-3p, miR-342, and miR-223-3p (Fig. 4D). These downregulated markers were only decreased at 12 Gy. Due to the low number of differentially expressed miRNA, all probes featuring a linear trend upwards or downwards are shown (Supplemental Table S3).

### Few lncRNA showed a continuous dysregulation in expression after WBI

**Figure 5:**
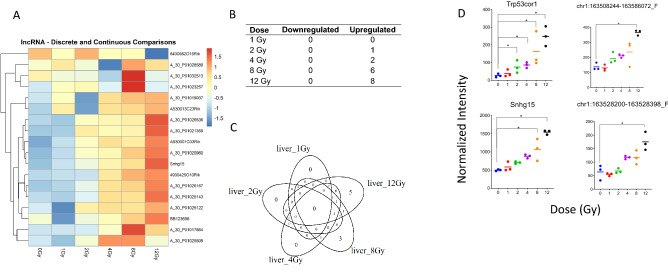
Radiation-induced long non-coding RNA expression profiles in mouse liver tissue. (**A**) Heatmap displays expression patterns, of all differentially expressed lncRNAs across all doses and controls. A linear model was fit to each lncRNA probe to assess differential expression of irradiated compared to control samples using criteria of |log2FC|> 1 and B-H *p* value < 0.05. (**B**) The table shows the number of discrete down- versus up-regulated lncRNAs at each dose. (**C**) Venn diagram shows dose distribution and overlap of differentially expressed lncRNAs across all doses. (**D**) Examples of significant linearly up- and down-regulated lncRNAs are shown to display the dose response of lncRNAs to radiation in liver tissue samples. Asterisk (*) indicates statistical significance.

To examine the relationship between lncRNA and WBI, we filtered whole genome data to only include probes associated with lncRNA. Both discrete and continuous differential expression of lncRNA is depicted in heatmap (Fig. 5A). Some lncRNA display non-monotonic expression with increasing doses of radiation, notably A_30_P01028589 and A_30_P01019037. (Fig. 5A). Discretely dysregulated lncRNA between any dose and 0 Gy samples are shown in Fig. 5B,C. The probes of interest were *Trp53cor1, Snhg15*, chr1:163528200-163528398_F and chr1:163508244-163586072_F, (Fig. 5D). No lncRNA were significant at 1 Gy (Fig. 5B,C). By 2 Gy, *Trp53cor1* had increased to a statistically significant degree and remained upregulated for the higher doses. By 8 Gy, 5 lncRNA including *Snhg15* were significantly upregulated. At 12 Gy there were 8 lncRNA which became statistically upregulated, including chr1:163528200-163528398_F and chr1:163508244-163586072_F. All lncRNA which show a significant linear dose response are presented in Supplemental Table S4.

### Significant genes found in microarray are validated and consistent across multiple strains

To ensure that significant gene expression changes in response to radiation were not specific to the C57BL/6 J mouse strain, an identical PCR analysis was conducted on C3H mice. The top upregulated genes (*Cdkn1a, Eda2r*, and *Phdla3*), as well as the most significantly downregulated gene (*Hba-a2*), were cross examined at 0, 1, 2, 4, and 8 Gy radiation dosages (Supplemental Fig. S4). Both C57BL/6 J and C3H showed significant upregulation in *Cdkn1a* at 2, 4, and 8 Gy. With regards to *Phlda3*, C3H began showing significant upregulation at 8 Gy, whereas *Phlda3* expression in C57BL/6 J increased significantly at 2, 4, and 8 Gy. Both strains showed significant downregulation of *Hba-a2* beginning at 1 Gy. The consistency in gene regulation post-radiation across multiple strains reinforces the promise of using biomarkers for radiation bio-dosimetry.

In contrast, non-coding RNA *Trp53cor1* and miR-34a did not display consistency between C57BL/6 J and C3H mice (Supplemental Fig. S5). *Trp53cor1* was statistically significant at 1, 2, and 8 Gy in C57BL/6 J, but was only significant at 4 Gy in C3H. Dino was statistically significant at 2, 4, and 8 Gy in both C57BL/6 J and C3H mice. The miR-34a was significant at 4 and 8 Gy in C57BL/6 J mice, but only reached significance at 4 Gy for C3H mice.

## Discussion

Radiation induced liver disease (RILD) limits the application of radiotherapy for the treatment of liver cancers^47–49^. Rescuing the normal liver function and prevention of long-term radiation toxicity demands understanding of the molecular changes induced by radiation in normal liver cells which could then be evaluated for therapeutic interventions.

We observed that some genes in our study have not previously been associated with radiation or any functions, including NR_045989, Gm45941, Gm41572, and Gm39334 (Supplemental Table S2, S5). In addition, some RNA have only received superficial attention for their role in radiation response including miR-8110 (Supplemental Table S4)^50^. Further information on their roles may lead to as yet undiscovered opportunities for mitigation or utilization as biomarkers for pathology. We recognize that not all genes showed linear dysregulation; Sult2a4 showed increased expression at 2, 4, and 8 Gy and decreased expression at 12 Gy. This non-monotonic response has previously been observed in other animal and human radiation research^51,52^. We focus the discussion on previously reported RNA and RNA relevant to specific pathway dysfunctions as those may serve as biomarkers of injury and of targets for injury mitigation.

### WBI dysregulates genes relevant to hemoglobin synthesis, radiation stress response, immune response and cell cycle arrest in the liver

Multiple hemoglobin family gene members, such as *Hbb-b1, Hbb-b2, Hbb-bt, Hba-a1*, and *Hba-a2*, were downregulated in the mouse liver after increasing doses of WBI. Increased levels of free hemoglobin in serum were linked to higher levels of hepatic steatosis, non-alcoholic fatty liver disease, and other metabolic disorders of the liver in males in human population studies^53–55^. Prior data shows hepatocytes synthesize hemoglobin to decrease oxidative stress^56^. This observed downregulation in our data may indicate damage to the hepatocytes.

The most dose–response upregulated genes were *Cdkn1a, Phlda3*, and *Eda2r*, which are regulated by p53 to induce cell cycle arrest or apoptosis^57^. All three have previously been highlighted as predictive markers of radiation injury in murine models^38,58–61^. *Cdkn1a* has previously been reported as a marker of irradiation in cancer patients undergoing WBI and in murine models from our lab and others^25,35,62^.

The liver has known functions in metabolism and xenobiotic detoxification. This detoxification includes an immunological response to viruses, bacteria, and other potential pathogens^63^. Among the most downregulated genes after radiation were two involved in B cell differentiation and activation: *Serpina9* and *Ms4a1*^64,65^. *Ms4a1* was one of the most downregulated genes in the blood of male prostate cancer patients suffering from fatigue after radiation therapy^39^. *Serpina9*, also known as centerin and GCET1, is found in germinal centers of B cells though its function is not well understood^65^. While these genes are associated with B cell differentiation, it is possible they also have as yet unelucidated roles in other cell types.

### Liver transport and detoxification are compromised after radiation injury

As shown in Fig. 3B, the *Slc* gene family codes for membrane proteins which allow passive, symport and antiport transport of amino acids, lipids, glucose, anions and cations across the membrane^66^. These solute carriers may be found on the cellular membrane or the membrane of various organelles and impact drug absorption^67^. Some are highly substrate specific, while others will transport a range of substrates across. For brevity, the roles of many of these *Slc* are noted here^67–76^.

Highlighted are the *Slc* most relevant to glucose and fatty acid metabolism. *Slc5a1* also called sodium-glucose cotransporter 1 (SGLT1) uses the sodium electrochemical gradient to move glucose into cells^77^. *Slc16a6* transports ketone bodies, while *Slc16a7* transports pyruvate, lactate, and ketone bodies^70,71^. Interestingly, some *Slc* transporters may act indirectly to modify metabolism, *Slc22a14* has previously been shown to indirectly impact triglyceride storage and fatty acid oxidation in a mouse model^78^.

Only two active transporters were dysregulated. Both *Abcd2* and *Abca8* are ATP Binding Cassette family members relevant to lipid metabolism^79,80^. While Very low density lipoprotein receptor (Vldlr) mediates lipid uptake and accumulation^81,82^. These changes may give insight into energy production pathways within the liver after radiation injury and potential biomarkers to understand liver damage and response over time.

Briefly, *Cyp2u1, Cyp2j9, Cyp4f18 and Cyp11b1* all function in arachidonic acid and cholesterol metabolism^83–87^. *Cyp26a1* and *Cyp26b1* regulate retinol^88^. Modification of these pathways has implications for inflammation and response to reactive oxygen species which are produced after radiation injury. *Cyp2u1* and *Cyp46a1* function in lipid storage and mitochondrial function, with downregulation associated with increased triglyceride synthesis and hepatic lipid droplet formation^84,89,90^ Radiation-induced dysregulation of cytochrome p450 expression can impact inflammation and metabolism leading to long term dysfunction if unrepaired. *Gsta2, Sult2*, and *Nqo1* aid in detoxification and efflux of metabolites and xenobiotics^91–94^. Dysregulation of these pathways has implications for efficacy of medications given post injury. In knowing that the liver has a reduced capacity to metabolize certain medications, clinicians must modify medication doses to avoid secondary toxic effects caused by this differential rate of medication metabolism.

### Liver energy homeostasis and lipid storage are dysregulated by radiation

Prior literature has shown that radiation induces increased triglyceride storage, lipid metabolism dysfunction, and mitochondrial dysfunction in the liver^10,95,96^. These studies led us to interrogate the impact of radiation on lipid metabolism in our mouse model. Briefly, in Fig. 4B, *Acaca, Acacb, Acss1, Acss2, Scd1, Scd3, Plin4, Plin5, Srebf1, Lipin1, and Lipi*n2 have functional roles in lipogenesis and triacylglycerol maintenance^97–102^.

Carnitine palmitoyl transferase 1 (Cpt1), Acyl-CoA dehydrogenase (Acad) and the mitochondrial trifunctional protein beta subunit (Hadhb) are known to bring fatty acids into the mitochondria, and perform needed steps for fatty acid oxidation^103–105^. Overall, we see a decrease in fatty acid uptake into the liver and a decrease in peroxisomal fatty acid oxidation as radiation doses increase. We also observe increased triacylglycerol synthesis and maintenance after 8 and 12 Gy doses of radiation. Prior research in rats indicate that selective 25 Gy radiation to the liver induced fat accumulation at 48 h post radiation^106^.

### Most significantly dysregulated miRNAs play role in glucose metabolism and inflammation

Interestingly, while IPA did not implicate glucose metabolism as a dysregulated canonical pathway after radiation, multiple significantly altered miRNA affect glucose metabolism. Prior research into dysregulation of miR-34a-5p, miR-3102-5p, and miR-142-3p demonstrate that their dysregulation encourages insulin resistance and decreased glucose metabolism^107–109^ While we did not see significant dysregulation of rate limiting genes in glucose oxidation. We do see dysregulation of *Slc5a1*, a protein co-transporter of glucose and sodium (Fig. 3A).

### Dysregulation of lncRNA may have implications for hemoglobin synthesis, and proliferation

While several lncRNA were shown to be dysregulated by radiation in our study, few have received even a name, and their function in normal tissue are poorly understood^110,111^. *Trp53cor1* knockdown in combination with radiation produced decreased apoptosis in mouse embryonic fibroblasts^112^. We have previously observed upregulation of this lncRNA and *Dino* in whole mouse blood and in mouse heart after radiation injury^28^.

### There was overlap in gene expression after radiation between Gottingen minipigs and mice indicating potential biomarkers

One limitation of our study is that we chose an early time point to observe genetic dysregulation. While we did not observe death at our short time point in the present mouse study, the anticipated LD50/60 for WBI of C57BL/6 J mice is approximately 7.69–7.81 Gy^113^. We have chosen to compare our early changes in our mice to our long-term study of Gottingen minipigs^114^. In that study, minipigs were followed for 45 days post-radiation. Animals were grouped as survivors and decedents depending on whether they survived till 45 days. We identified survival-predictive RNA biomarkers of liver injury in these minipigs. Interestingly, Serum Amyloid A2 (Saa2) was upregulated in the liver of decedent mini pigs. We observed a similar significant upregulation of *Saa2* in our mouse liver for mice irradiated at 8 and 12 Gy compared to controls (Supplemental Table S1). Similarly, *Gdf15* was upregulated in non-surviving pigs compared to survivors and controls. *Gdf15* was upregulated in mice at 12 Gy compared to controls (Supplemental Table S1). Several metabolism markers were downregulated in non-survivors compared to survivors including Phosphoglycerate dehydrogenase (Phgdh), *Acss2*, and *Scd1*. *Phgdh* and *Acss2* were only downregulated in mice at 8 Gy compared to controls, while *Scd1* was downregulated in mice at 8 and 12 Gy compared to controls (Supplemental Table S1). This similarity in gene expression across species suggests potential for these genes to be used as part of an RNA panel of radiation biomarkers.

### RNA biomarkers must be carefully selected

Some RNAs may be good general indicators of radiation exposure while others may be more organ specific and suggest targets for mitigation. The use of miR-34a is an example: differences in age, diabetes status and radiation are all known to impact miR-34a expression^115–117^. Similarly, pathophysiological differences between human and animal model response to radiation highlight potential obstacles in developing an RNA biomarker panel. While animal models provide useful initial information, radiation-induced liver disease in the mouse does not present with veno-occlusive lesions, which is a hallmark in human patients with RILD^17,118^. To validate the RNA and pathway dysregulation observed in this study and to create useful RNA panels for human patients, our lab will next focus on 3D cultures using multiple human primary cell types together to recapitulate normal human organ response to radiation damage.

This experiment was an initial study to demonstrate the potential utility of lncRNA-miRNA-mRNA in a biomarker panel to determine radiation biodosimetry and elucidate dysregulated pathways to aid in clinical triage and medical decision making. Other types of RNA including piRNA, tsRNA and rsRNAs are receiving attention^119,120^ and should be explored for their utility in building these panels as well.

### Future directions

Understanding the expected patterns of radiation-induced early gene dysregulation in the liver and the dose–response pattern will be useful for diagnosing and mitigating RILD following whole body exposure. It may aid in clinical management of radiotherapy patients. With potential metabolic targets of injury indicated, including dose–response relationships, ongoing studies will address potential mitigators.

## Methods

### Total body irradiation of mice and sample collection

Six- to eight-week-old female C57BL/6 J and C3H mice received whole body irradiation (WBI) with x-rays using the Small Animal Radiation Research Platform (SARRP Xstrahl Ltd.). Mice were placed in plastic containers and exposed to a single surface dose of 1, 2, 4, 8, or 12 Gy at a dose rate of 1.05 Gy/min. Control mice (0 Gy) were placed in the same type of plastic container and sham irradiated. Three animals per dose were included in the study. Livers of irradiated and control animals were harvested 48 h after WBI. Organs were snap frozen in liquid nitrogen and stored at  − 80 °C until processed for RNA isolation. The experimental protocol was approved by a New York University (NYU) Langone Medical Center under an approved IACUC protocol as part of a collaborative study. Tissue collection validation studies were performed at the National Cancer Institute, Radiation Oncology branch using a Pantak x-ray source at a dose rate of 2.28 Gy/min and conducted in accordance with the principles and procedures outlined in the NIH Guide for the Care and Use of Animals and procedures. All methods are reported in accordance with ARRIVE guidelines (https://arriveguidelines.org).

### RNA isolation

Samples were bathed in liquid nitrogen and pulverized into a fine powder using a mortar and pestle. Approximately 100 µg of powdered sample was lysed with 700 µl of QIAzol lysis buffer (Cat # 79306, QIAGEN) and homogenized by passing the solution through QIAshredder spin columns (Cat # 79654, QIAGEN). RNA isolation was performed using standard miRNeasy mini kit (Cat # 217004, QIAGEN) according to the manufacturer’s protocol. Quality and quantity of the RNA samples were assessed using a DeNovix DS-11 nanodrop spectrophotometer (DeNovix, DE, US) and Agilent Bioanalyzer with the RNA6000 Nano Lab Chip (Agilent Technologies, Santa Clara, CA).

### Microarray analysis

Microarray analysis was performed for sham animals (0 Gy) and 1 Gy, 2 Gy, 4 Gy, 8 Gy, and 12 Gy irradiated animals. Quality assessments and microarray experiments were completed as previously reported^33^. Samples were hybridized to Agilent Mouse GE 8×60 K v2 arrays for mRNA expression analysis and to Agilent Mouse miRNA 8×60 K v21.0 arrays (Design ID 070155) for miRNA expression analysis. Slides were washed and scanned on an Agilent SureScan Microarray Scanner. Expression values were extracted using Agilent Feature Extraction software.

### Real time RT-PCR analysis of RNAs

Individual qRT-PCR reactions using RT2 qPCR primer assays along with RT2 First Strand Synthesis kit and RT2 SYBR Green qPCR Master Mix (QIAGEN) were performed. The following RNA primers were purchased from Qiagen, gene globe IDs are included for mRNA and assay IDs for non-coding RNA: Cdkn1a (PPM02901B-200), Eda2r (PPM32677A-200), Phlda3 (PPM28194A-200), Hba-a2 (PPM69448A-200), miR-34a (YP00204486), Trp53cor1 (LPM12776A), Dino^26^(FP- GCAATGGTGTGCCTGACTAT; RP- TTCTGGCTTCCCAGAG), and Rplp0 (PPM03561B). QRT-PCR analysis was performed on select miRNA, lncRNA, and mRNA to validate results and determine cross-strain accuracy as C3H mouse liver was also tested for RNA expression. C3H organ RNA extraction and qRT-PCR analysis were the same as explained above. Relative expression was calculated as: 2^−dCt^ where dCt = Ct [test gene] − Ct Rplp0^28^.

### Statistical analysis

Analysis of mRNA and miRNA data was performed using R statistical software and the Bioconductor Linear Model for Microarray Analysis (LIMMA) package in R^121^. Background correction and normalization were performed in R using the normal-exponential correction method and quantile normalization between arrays^122^. Only probes with intensities above background on at least one array were kept in the dataset for analysis. Transcripts with multiple probes were averaged such that the final set reflected best estimates of transcript level expression. A linear model was fit to each probe to assess differential expression for pair-wise dose comparisons within the liver-tissue samples. This method employed an empirical Bayes smoothing approach that results in more stable model estimates by using information on variance from the whole probe set, despite the small number of arrays. Models were developed for each of the pair-wise comparisons between each dose (1, 2, 4, 8, and 12 Gy) and the control probes (0 Gy), and resulting probes were filtered using log_2_ fold change and adjusted *p* value thresholds (|log_2_FC|> 1, adjusted *p* value < 0.05)^123^. Additionally, a nested interaction model was fit for each probe to examine dose within tissue as a linear (continuous) trend. Each model yielded the main effects for the liver tissue and dose within the liver tissue. Probes were filtered using the nested dose coefficients with log fold change and adjusted *p* value thresholds (|log_2_FC|> 1, adjusted *p* value < 0.05).

To identify potential interactions, paired analysis was conducted to evaluate correlative relationships between pairs of differentially expressed mRNA and miRNA probes. mRNA and miRNA probes were paired using shared target transcript Ensembl IDs^124^. Probes that could not be mapped or paired were excluded. Transcripts for miRNA probes were identified using an Agilent microarray gene dataset and the TargetScan database; transcripts for mRNA probes were identified using an Agilent microarray gene dataset^125^. Transcript-miRNA pairs with a TargetScan context++ score above  − 1 were excluded. Probe pairs with differentially expressed miRNA and mRNA probes were identified within the liver tissue for continuous dose contrast models. Pearson correlation coefficients of miRNA and mRNA expression across all experiments were calculated and plotted for the differentially expressed probe pairs.

### Ingenuity pathway analysis

Both core and comparison analyses were performed in IPA (QIAGEN Inc., https://www.qiagenbioinformatics.com/products/ingenuitypathway-analysis). Pathways and function terms that satisfied an absolute z-score > 2 and *p* value < 0.01 were predicted to be altered based on the gene expression data.

### Ethics approval and consent to participate

The experimental protocol was approved by a New York University (NYU) Langone Medical Center under an approved IACUC protocol as part of a collaborative study.

## Supplementary Information

Supplementary Information 1.

Supplementary Information 2.

Supplementary Information 3.

Supplementary Information 4.

## Data Availability

Data is available at NCBI GEO #GSE202586.

## Abbreviations

RT: Radiotherapy
IR: Ionizing radiation
miRNAs: MicroRNAs
lncRNAs: Long non-coding RNAs
mRNAs: Messenger RNAs
WBI: Whole body irradiation
RILD: Radiation induced liver disease
