# Supplementary material for: Microarray analysis identifies coding and non-coding RNA markers of liver injury in whole body irradiated mice
Source: Sci Rep. 2023 Jan 5;13:200. doi: 10.1038/s41598-022-26784-w (PMC9814510; doi:10.1038/s41598-022-26784-w)
Supplement: Supplementary file 1 — Supplementary Information 1. [file 41598_2022_26784_MOESM1_ESM.pptx]

## Slide 1
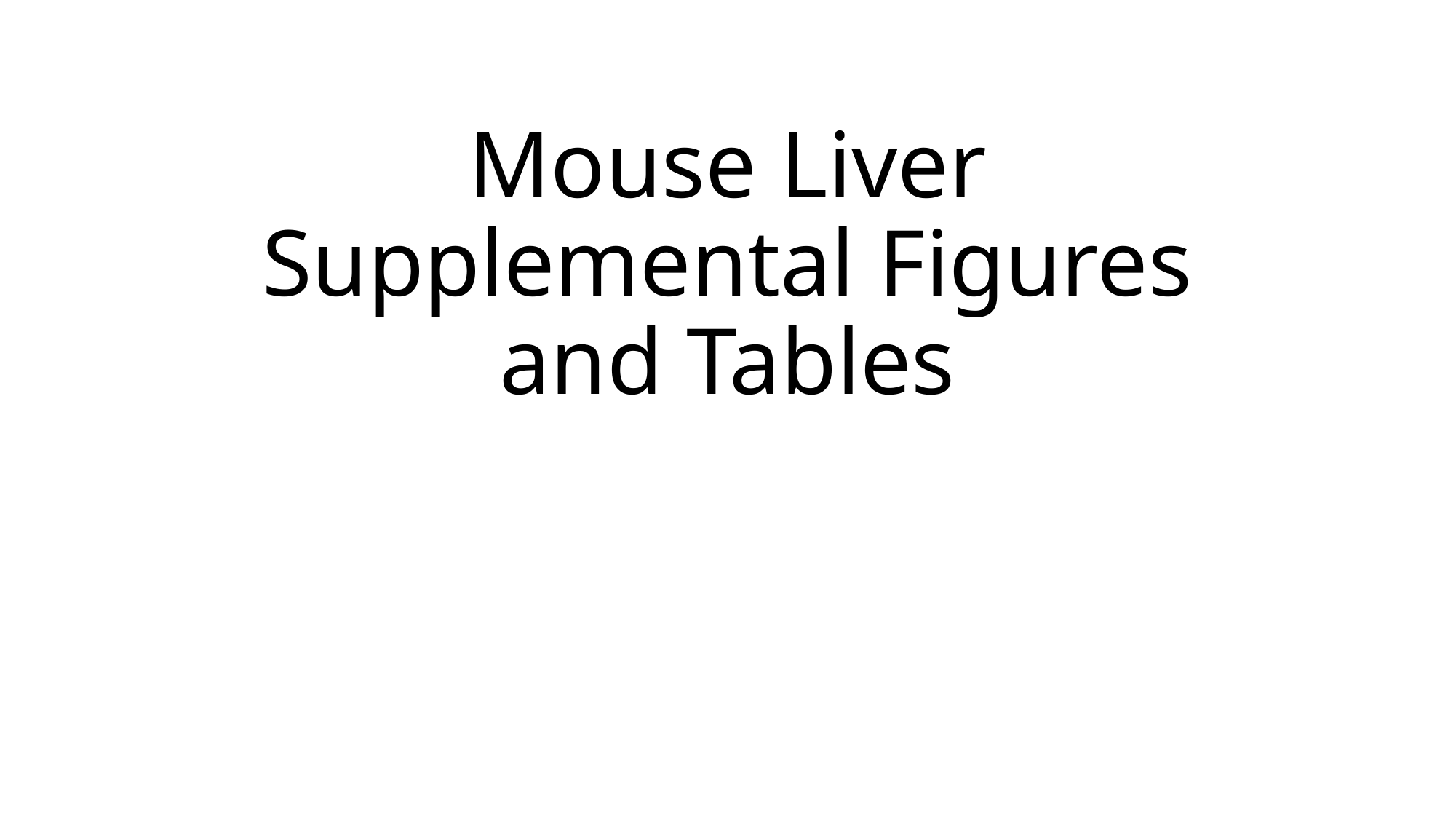

# Mouse Liver Supplemental Figures and Tables

## Slide 2
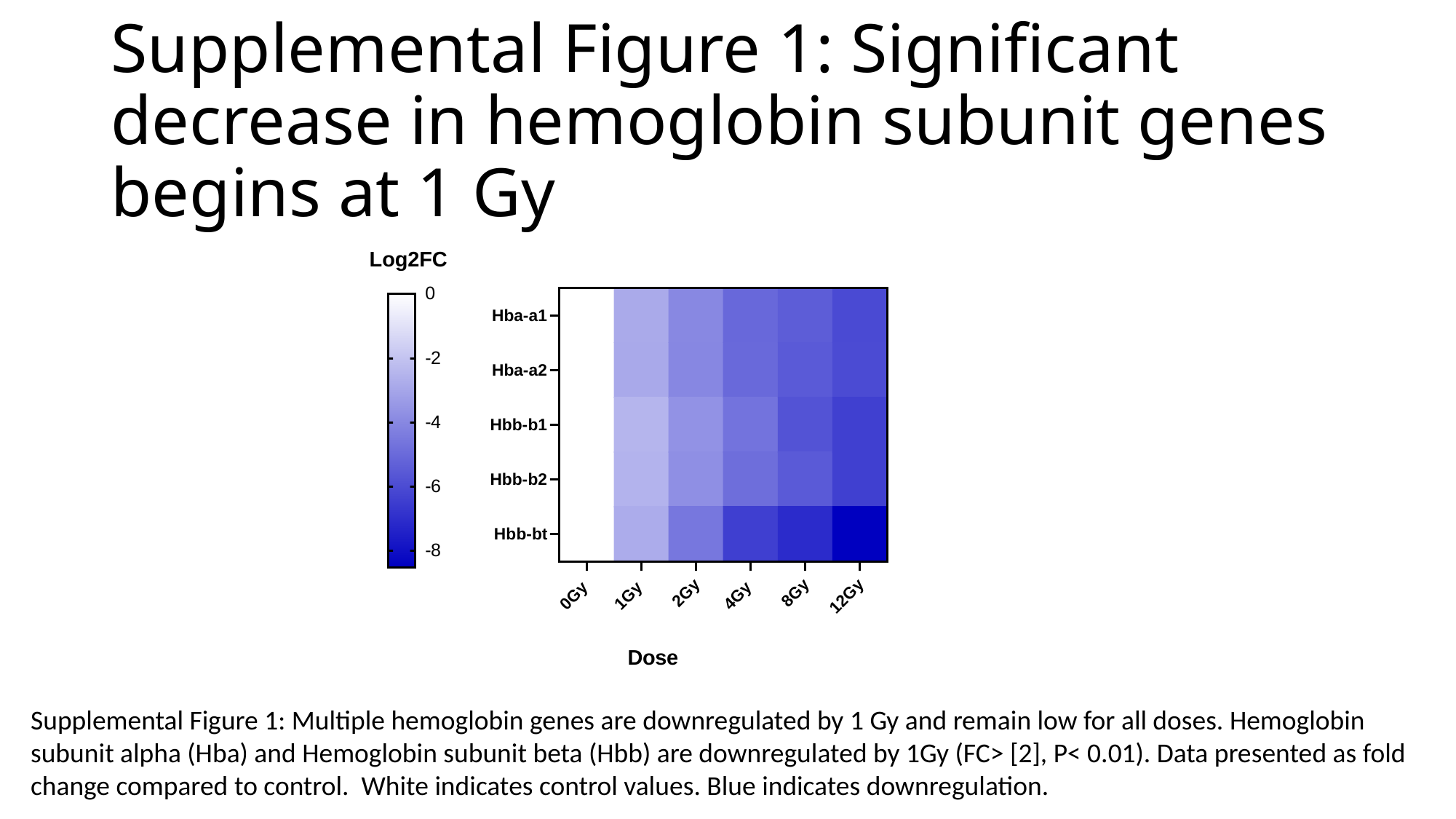

# Supplemental Figure 1: Significant decrease in hemoglobin subunit genes begins at 1 Gy
Supplemental Figure 1: Multiple hemoglobin genes are downregulated by 1 Gy and remain low for all doses. Hemoglobin subunit alpha (Hba) and Hemoglobin subunit beta (Hbb) are downregulated by 1Gy (FC> [2], P< 0.01). Data presented as fold change compared to control. White indicates control values. Blue indicates downregulation.

## Slide 3
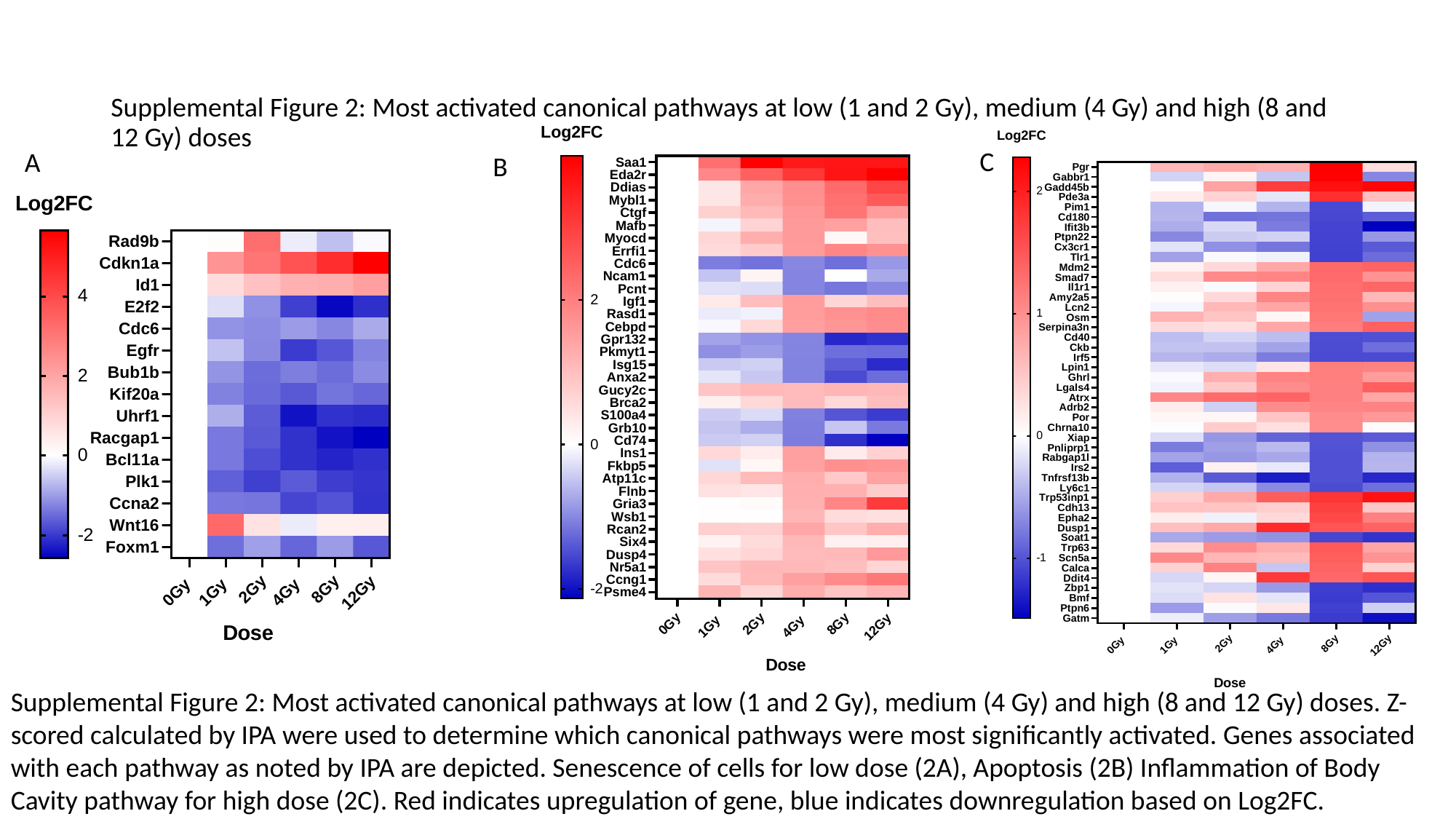

# Supplemental Figure 2: Most activated canonical pathways at low (1 and 2 Gy), medium (4 Gy) and high (8 and 12 Gy) doses
C
A
B
Supplemental Figure 2: Most activated canonical pathways at low (1 and 2 Gy), medium (4 Gy) and high (8 and 12 Gy) doses. Z-scored calculated by IPA were used to determine which canonical pathways were most significantly activated. Genes associated with each pathway as noted by IPA are depicted. Senescence of cells for low dose (2A), Apoptosis (2B) Inflammation of Body Cavity pathway for high dose (2C). Red indicates upregulation of gene, blue indicates downregulation based on Log2FC.

## Slide 4
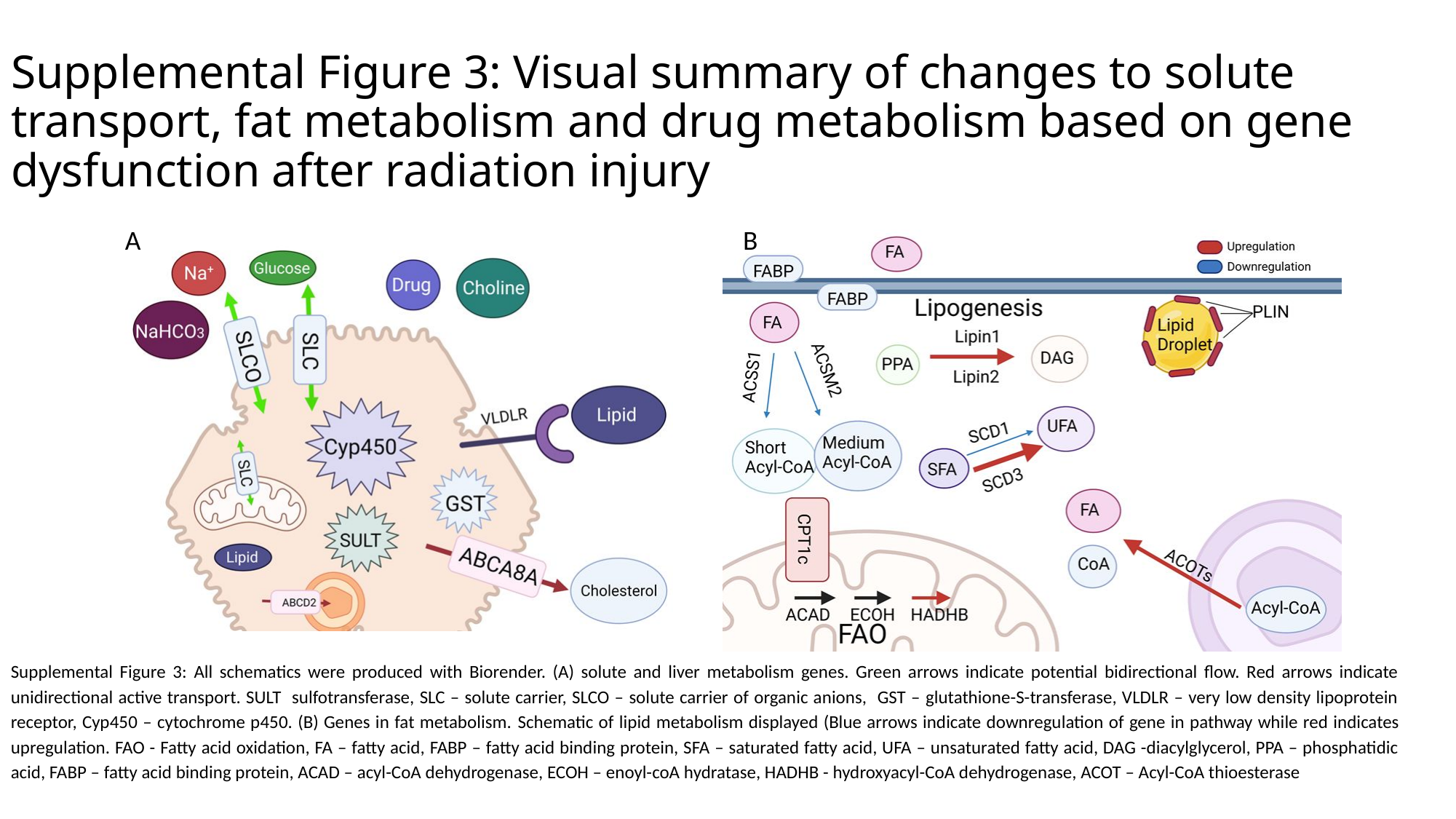

# Supplemental Figure 3: Visual summary of changes to solute transport, fat metabolism and drug metabolism based on gene dysfunction after radiation injury
A
B
Supplemental Figure 3: All schematics were produced with Biorender. (A) solute and liver metabolism genes. Green arrows indicate potential bidirectional flow. Red arrows indicate unidirectional active transport. SULT sulfotransferase, SLC – solute carrier, SLCO – solute carrier of organic anions, GST – glutathione-S-transferase, VLDLR – very low density lipoprotein receptor, Cyp450 – cytochrome p450. (B) Genes in fat metabolism. Schematic of lipid metabolism displayed (Blue arrows indicate downregulation of gene in pathway while red indicates upregulation. FAO - Fatty acid oxidation, FA – fatty acid, FABP – fatty acid binding protein, SFA – saturated fatty acid, UFA – unsaturated fatty acid, DAG -diacylglycerol, PPA – phosphatidic acid, FABP – fatty acid binding protein, ACAD – acyl-CoA dehydrogenase, ECOH – enoyl-coA hydratase, HADHB - hydroxyacyl-CoA dehydrogenase, ACOT – Acyl-CoA thioesterase

## Slide 5
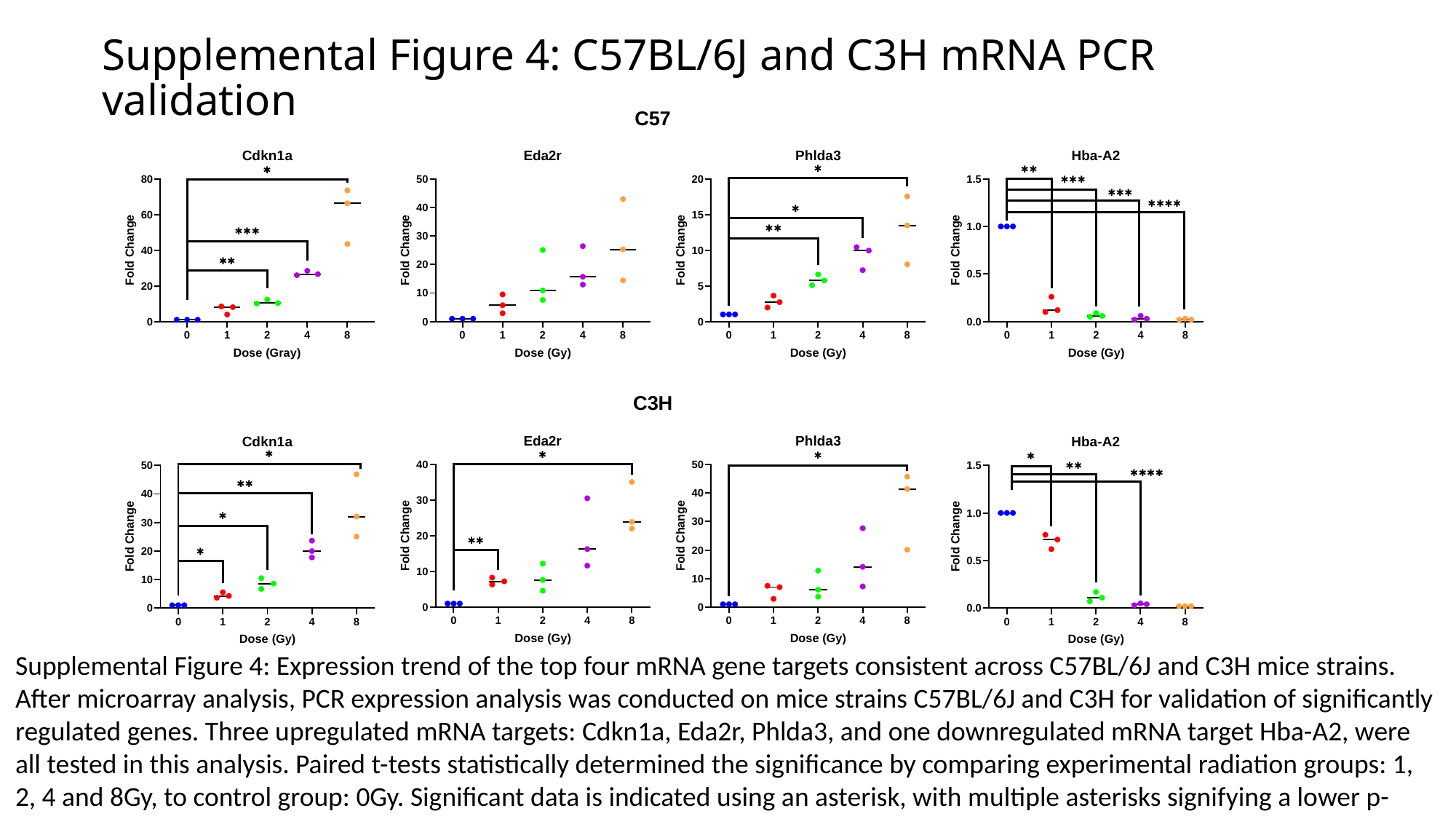

# Supplemental Figure 4: C57BL/6J and C3H mRNA PCR validation
Supplemental Figure 4: Expression trend of the top four mRNA gene targets consistent across C57BL/6J and C3H mice strains. After microarray analysis, PCR expression analysis was conducted on mice strains C57BL/6J and C3H for validation of significantly regulated genes. Three upregulated mRNA targets: Cdkn1a, Eda2r, Phlda3, and one downregulated mRNA target Hba-A2, were all tested in this analysis. Paired t-tests statistically determined the significance by comparing experimental radiation groups: 1, 2, 4 and 8Gy, to control group: 0Gy. Significant data is indicated using an asterisk, with multiple asterisks signifying a lower p-value.

## Slide 6
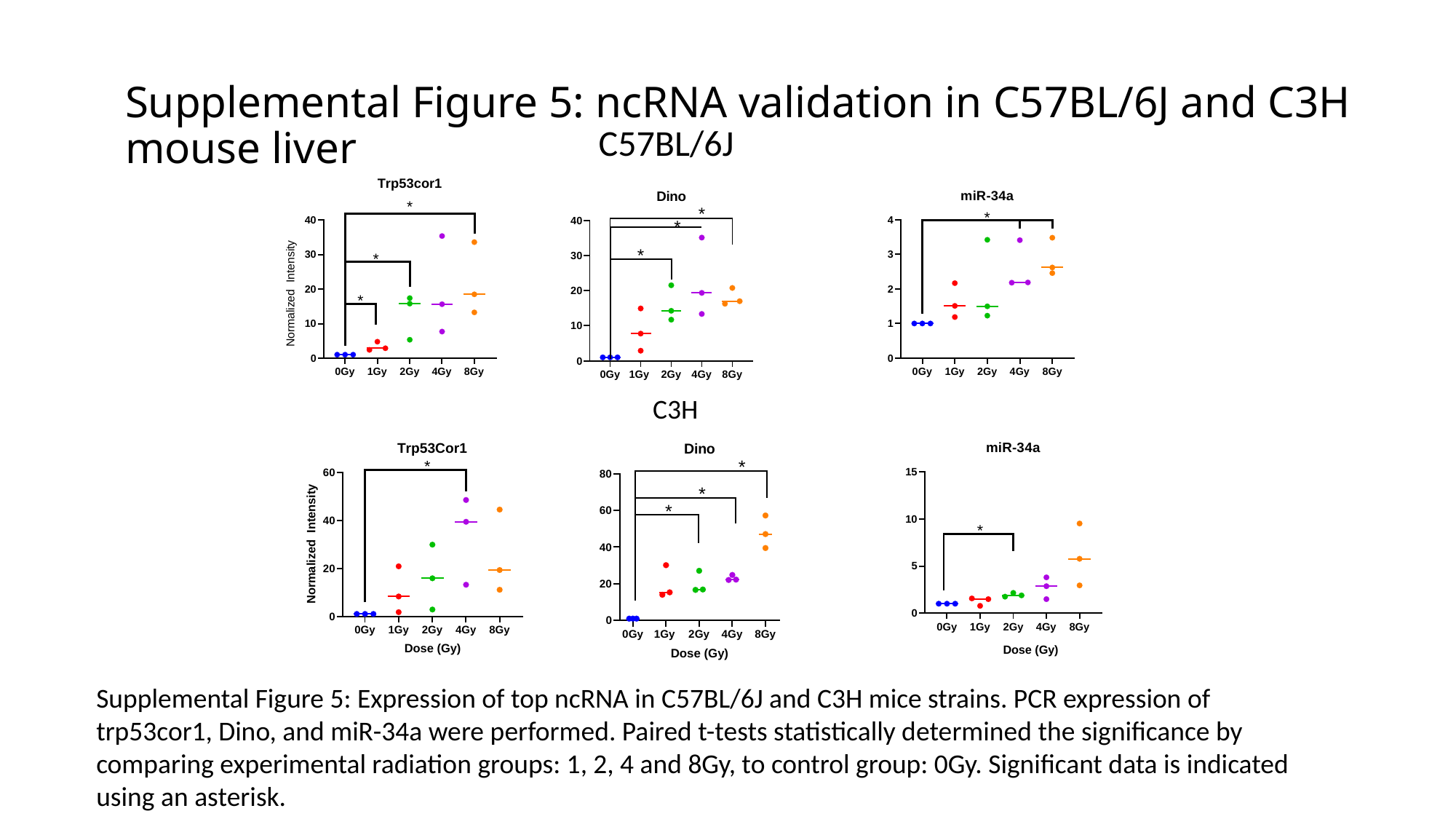

# Supplemental Figure 5: ncRNA validation in C57BL/6J and C3H mouse liver
C57BL/6J
C3H
Supplemental Figure 5: Expression of top ncRNA in C57BL/6J and C3H mice strains. PCR expression of trp53cor1, Dino, and miR-34a were performed. Paired t-tests statistically determined the significance by comparing experimental radiation groups: 1, 2, 4 and 8Gy, to control group: 0Gy. Significant data is indicated using an asterisk.

## Slide 7
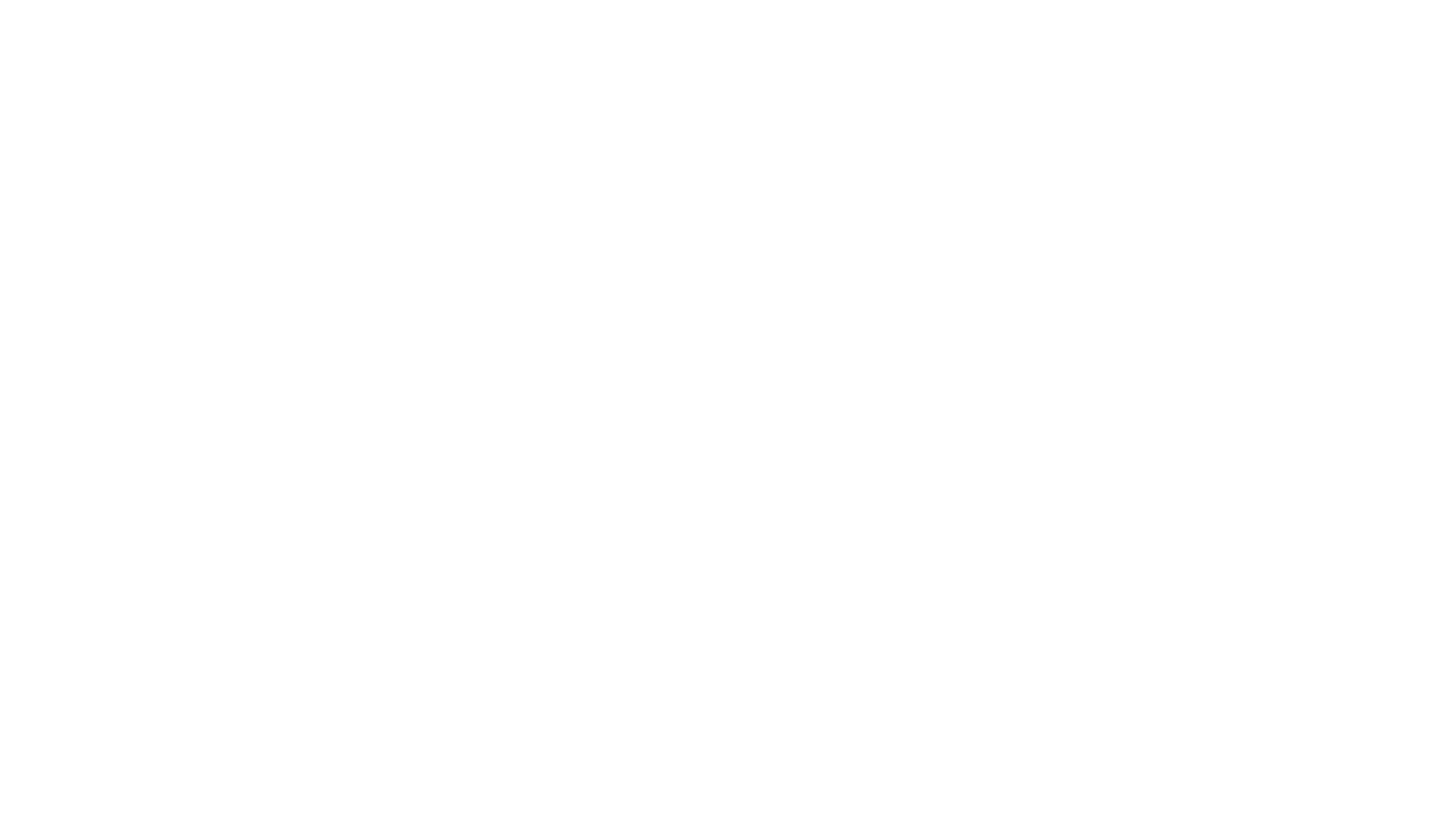

#
