# Supplementary material for: Microarray analysis identifies coding and non-coding RNA markers of liver injury in whole body irradiated mice
Source: Sci Rep. 2023 Jan 5;13:200. doi: 10.1038/s41598-022-26784-w (PMC9814510; doi:10.1038/s41598-022-26784-w)
Supplement: Supplementary file 2 — Supplementary Information 2. [file 41598_2022_26784_MOESM2_ESM.pptx]

## Slide 1
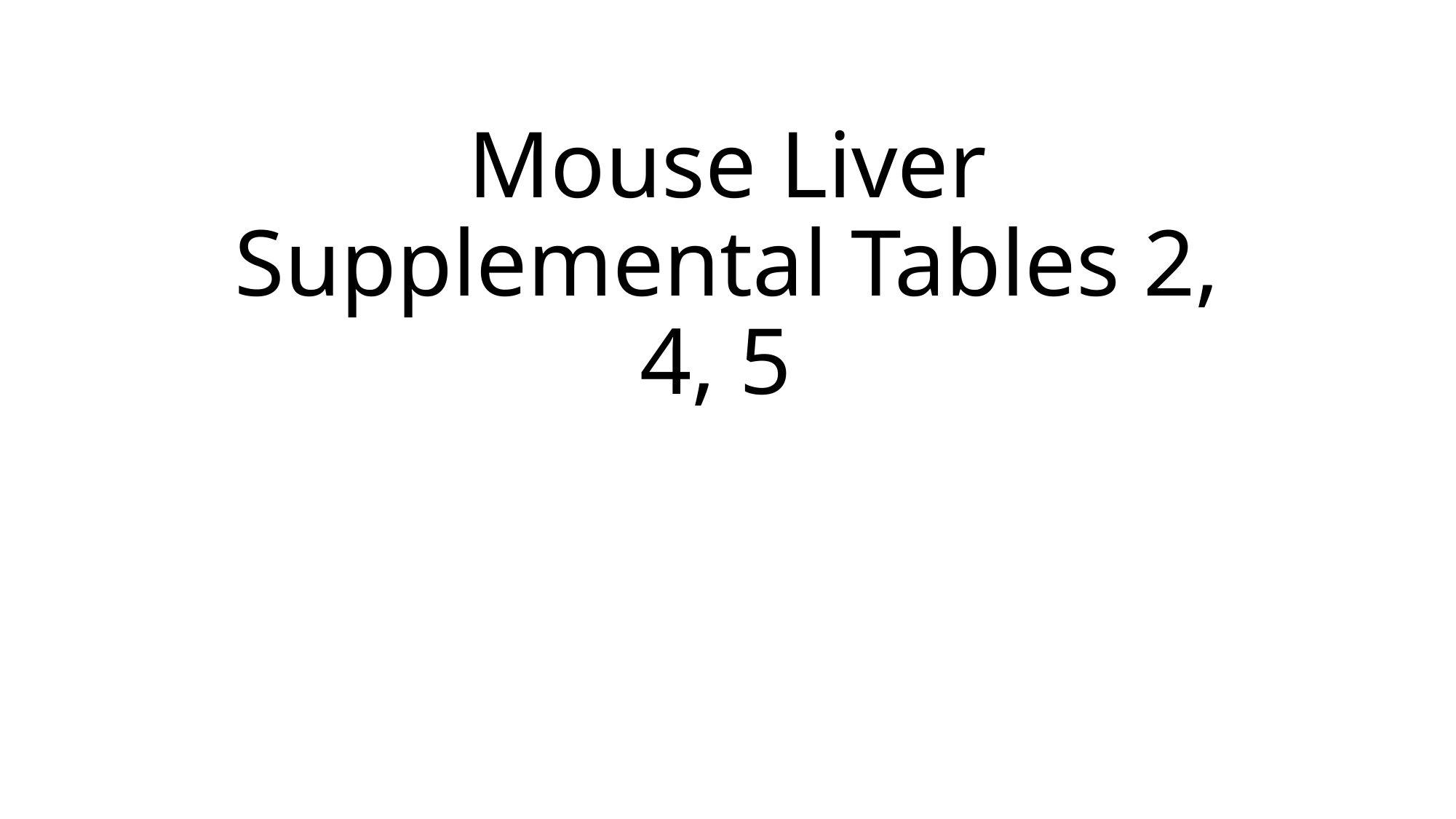

# Mouse Liver Supplemental Tables 2, 4, 5

## Slide 2
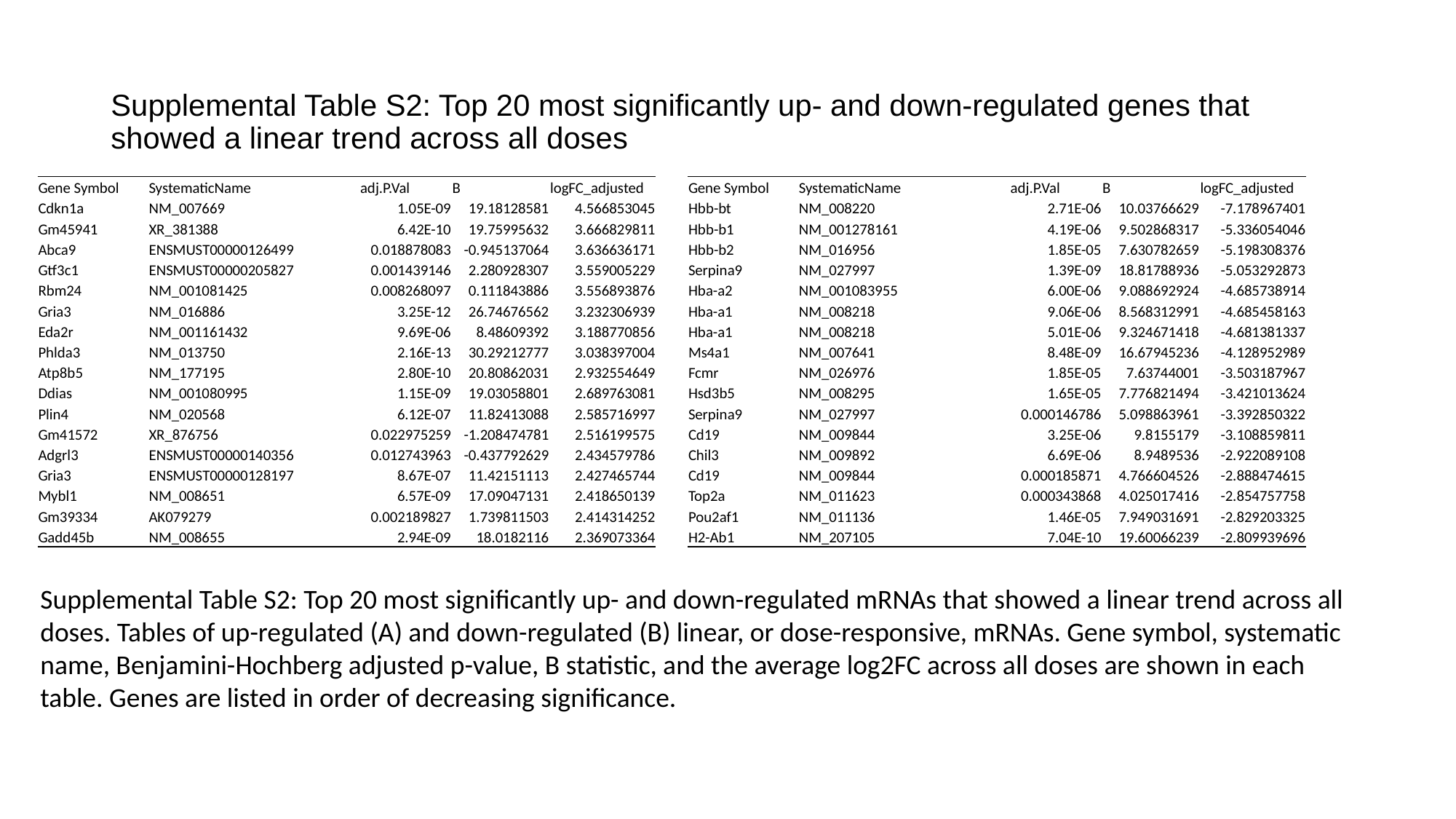

# Supplemental Table S2: Top 20 most significantly up- and down-regulated genes that showed a linear trend across all doses
| Gene Symbol | SystematicName | adj.P.Val | B | logFC\_adjusted |
| --- | --- | --- | --- | --- |
| Cdkn1a | NM\_007669 | 1.05E-09 | 19.18128581 | 4.566853045 |
| Gm45941 | XR\_381388 | 6.42E-10 | 19.75995632 | 3.666829811 |
| Abca9 | ENSMUST00000126499 | 0.018878083 | -0.945137064 | 3.636636171 |
| Gtf3c1 | ENSMUST00000205827 | 0.001439146 | 2.280928307 | 3.559005229 |
| Rbm24 | NM\_001081425 | 0.008268097 | 0.111843886 | 3.556893876 |
| Gria3 | NM\_016886 | 3.25E-12 | 26.74676562 | 3.232306939 |
| Eda2r | NM\_001161432 | 9.69E-06 | 8.48609392 | 3.188770856 |
| Phlda3 | NM\_013750 | 2.16E-13 | 30.29212777 | 3.038397004 |
| Atp8b5 | NM\_177195 | 2.80E-10 | 20.80862031 | 2.932554649 |
| Ddias | NM\_001080995 | 1.15E-09 | 19.03058801 | 2.689763081 |
| Plin4 | NM\_020568 | 6.12E-07 | 11.82413088 | 2.585716997 |
| Gm41572 | XR\_876756 | 0.022975259 | -1.208474781 | 2.516199575 |
| Adgrl3 | ENSMUST00000140356 | 0.012743963 | -0.437792629 | 2.434579786 |
| Gria3 | ENSMUST00000128197 | 8.67E-07 | 11.42151113 | 2.427465744 |
| Mybl1 | NM\_008651 | 6.57E-09 | 17.09047131 | 2.418650139 |
| Gm39334 | AK079279 | 0.002189827 | 1.739811503 | 2.414314252 |
| Gadd45b | NM\_008655 | 2.94E-09 | 18.0182116 | 2.369073364 |
| Gene Symbol | SystematicName | adj.P.Val | B | logFC\_adjusted |
| --- | --- | --- | --- | --- |
| Hbb-bt | NM\_008220 | 2.71E-06 | 10.03766629 | -7.178967401 |
| Hbb-b1 | NM\_001278161 | 4.19E-06 | 9.502868317 | -5.336054046 |
| Hbb-b2 | NM\_016956 | 1.85E-05 | 7.630782659 | -5.198308376 |
| Serpina9 | NM\_027997 | 1.39E-09 | 18.81788936 | -5.053292873 |
| Hba-a2 | NM\_001083955 | 6.00E-06 | 9.088692924 | -4.685738914 |
| Hba-a1 | NM\_008218 | 9.06E-06 | 8.568312991 | -4.685458163 |
| Hba-a1 | NM\_008218 | 5.01E-06 | 9.324671418 | -4.681381337 |
| Ms4a1 | NM\_007641 | 8.48E-09 | 16.67945236 | -4.128952989 |
| Fcmr | NM\_026976 | 1.85E-05 | 7.63744001 | -3.503187967 |
| Hsd3b5 | NM\_008295 | 1.65E-05 | 7.776821494 | -3.421013624 |
| Serpina9 | NM\_027997 | 0.000146786 | 5.098863961 | -3.392850322 |
| Cd19 | NM\_009844 | 3.25E-06 | 9.8155179 | -3.108859811 |
| Chil3 | NM\_009892 | 6.69E-06 | 8.9489536 | -2.922089108 |
| Cd19 | NM\_009844 | 0.000185871 | 4.766604526 | -2.888474615 |
| Top2a | NM\_011623 | 0.000343868 | 4.025017416 | -2.854757758 |
| Pou2af1 | NM\_011136 | 1.46E-05 | 7.949031691 | -2.829203325 |
| H2-Ab1 | NM\_207105 | 7.04E-10 | 19.60066239 | -2.809939696 |
Supplemental Table S2: Top 20 most significantly up- and down-regulated mRNAs that showed a linear trend across all doses. Tables of up-regulated (A) and down-regulated (B) linear, or dose-responsive, mRNAs. Gene symbol, systematic name, Benjamini-Hochberg adjusted p-value, B statistic, and the average log2FC across all doses are shown in each table. Genes are listed in order of decreasing significance.

## Slide 3
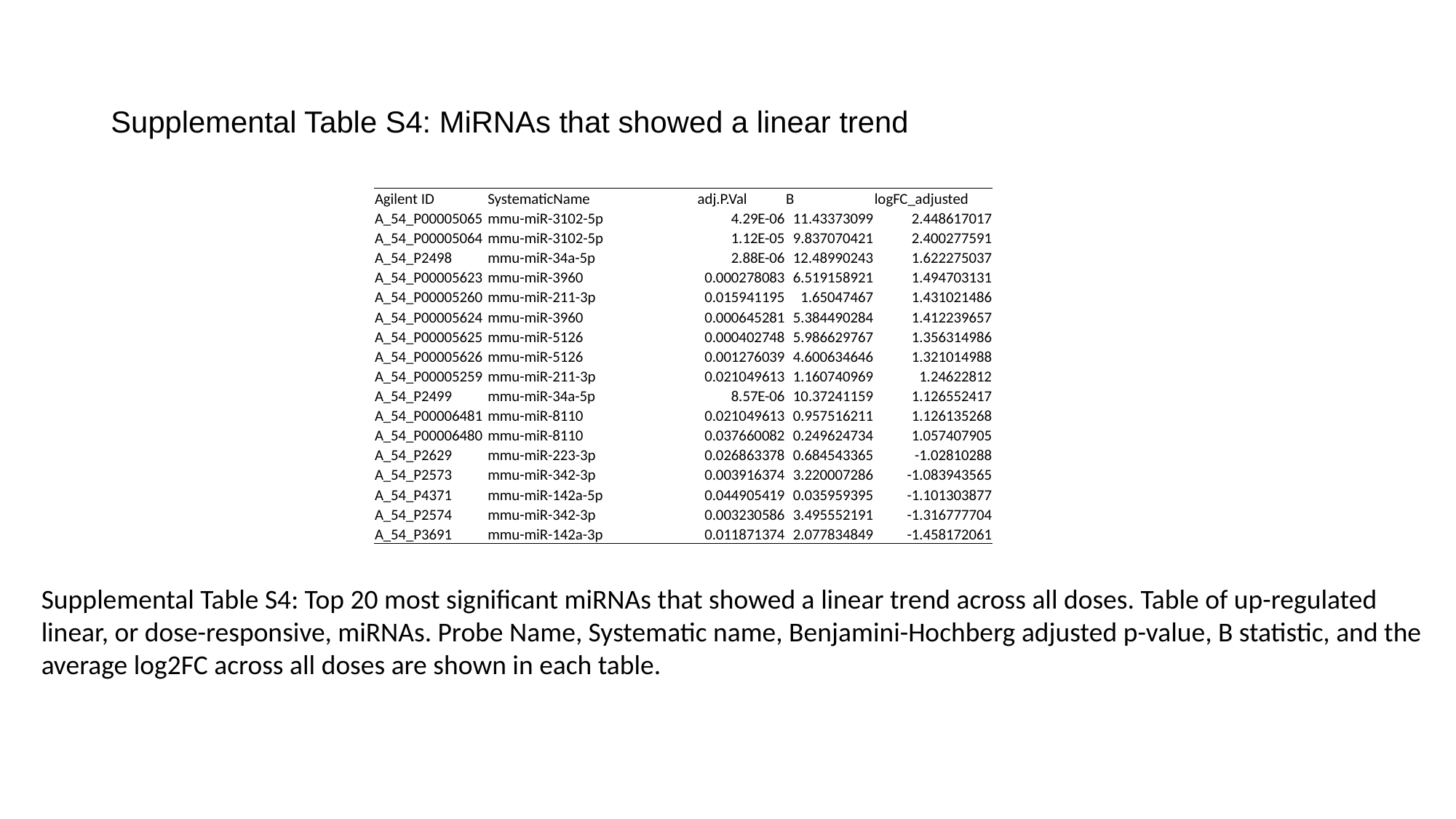

# Supplemental Table S4: MiRNAs that showed a linear trend
| Agilent ID | SystematicName | adj.P.Val | B | logFC\_adjusted |
| --- | --- | --- | --- | --- |
| A\_54\_P00005065 | mmu-miR-3102-5p | 4.29E-06 | 11.43373099 | 2.448617017 |
| A\_54\_P00005064 | mmu-miR-3102-5p | 1.12E-05 | 9.837070421 | 2.400277591 |
| A\_54\_P2498 | mmu-miR-34a-5p | 2.88E-06 | 12.48990243 | 1.622275037 |
| A\_54\_P00005623 | mmu-miR-3960 | 0.000278083 | 6.519158921 | 1.494703131 |
| A\_54\_P00005260 | mmu-miR-211-3p | 0.015941195 | 1.65047467 | 1.431021486 |
| A\_54\_P00005624 | mmu-miR-3960 | 0.000645281 | 5.384490284 | 1.412239657 |
| A\_54\_P00005625 | mmu-miR-5126 | 0.000402748 | 5.986629767 | 1.356314986 |
| A\_54\_P00005626 | mmu-miR-5126 | 0.001276039 | 4.600634646 | 1.321014988 |
| A\_54\_P00005259 | mmu-miR-211-3p | 0.021049613 | 1.160740969 | 1.24622812 |
| A\_54\_P2499 | mmu-miR-34a-5p | 8.57E-06 | 10.37241159 | 1.126552417 |
| A\_54\_P00006481 | mmu-miR-8110 | 0.021049613 | 0.957516211 | 1.126135268 |
| A\_54\_P00006480 | mmu-miR-8110 | 0.037660082 | 0.249624734 | 1.057407905 |
| A\_54\_P2629 | mmu-miR-223-3p | 0.026863378 | 0.684543365 | -1.02810288 |
| A\_54\_P2573 | mmu-miR-342-3p | 0.003916374 | 3.220007286 | -1.083943565 |
| A\_54\_P4371 | mmu-miR-142a-5p | 0.044905419 | 0.035959395 | -1.101303877 |
| A\_54\_P2574 | mmu-miR-342-3p | 0.003230586 | 3.495552191 | -1.316777704 |
| A\_54\_P3691 | mmu-miR-142a-3p | 0.011871374 | 2.077834849 | -1.458172061 |
Supplemental Table S4: Top 20 most significant miRNAs that showed a linear trend across all doses. Table of up-regulated linear, or dose-responsive, miRNAs. Probe Name, Systematic name, Benjamini-Hochberg adjusted p-value, B statistic, and the average log2FC across all doses are shown in each table.

## Slide 4
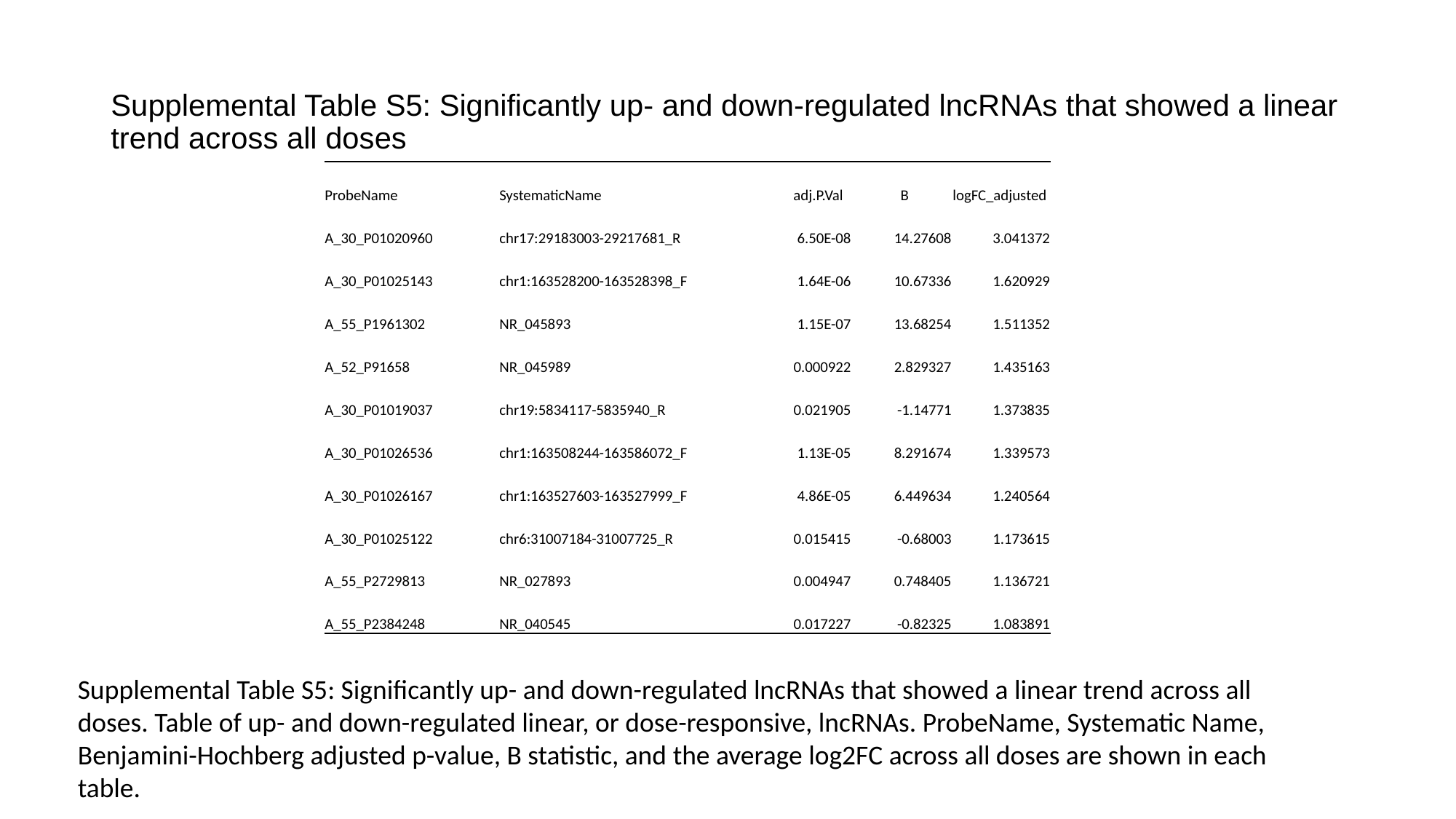

# Supplemental Table S5: Significantly up- and down-regulated lncRNAs that showed a linear trend across all doses
| ProbeName | SystematicName | adj.P.Val | B | logFC\_adjusted |
| --- | --- | --- | --- | --- |
| A\_30\_P01020960 | chr17:29183003-29217681\_R | 6.50E-08 | 14.27608 | 3.041372 |
| A\_30\_P01025143 | chr1:163528200-163528398\_F | 1.64E-06 | 10.67336 | 1.620929 |
| A\_55\_P1961302 | NR\_045893 | 1.15E-07 | 13.68254 | 1.511352 |
| A\_52\_P91658 | NR\_045989 | 0.000922 | 2.829327 | 1.435163 |
| A\_30\_P01019037 | chr19:5834117-5835940\_R | 0.021905 | -1.14771 | 1.373835 |
| A\_30\_P01026536 | chr1:163508244-163586072\_F | 1.13E-05 | 8.291674 | 1.339573 |
| A\_30\_P01026167 | chr1:163527603-163527999\_F | 4.86E-05 | 6.449634 | 1.240564 |
| A\_30\_P01025122 | chr6:31007184-31007725\_R | 0.015415 | -0.68003 | 1.173615 |
| A\_55\_P2729813 | NR\_027893 | 0.004947 | 0.748405 | 1.136721 |
| A\_55\_P2384248 | NR\_040545 | 0.017227 | -0.82325 | 1.083891 |
Supplemental Table S5: Significantly up- and down-regulated lncRNAs that showed a linear trend across all doses. Table of up- and down-regulated linear, or dose-responsive, lncRNAs. ProbeName, Systematic Name, Benjamini-Hochberg adjusted p-value, B statistic, and the average log2FC across all doses are shown in each table.
